# Kami-shoyo-san improves ASD-like behaviors caused by decreasing allopregnanolone biosynthesis in an SKF mouse model of autism

**DOI:** 10.1371/journal.pone.0211266

**Published:** 2019-01-31

**Authors:** Qing-Yun Guo, Ken Ebihara, Takafumi Shimodaira, Hironori Fujiwara, Kazufumi Toume, Dya Fita Dibwe, Suresh Awale, Ryota Araki, Takeshi Yabe, Kinzo Matsumoto

**Affiliations:** 1 Institute of Natural Medicine, University of Toyama, Toyama, Japan; 2 Laboratory of Functional Biomolecules and Chemical Pharmacology, Faculty of Pharmaceutical Sciences, Setsunan University, Osaka, Japan; Hudson Institute, AUSTRALIA

## Abstract

Dysfunctions in the GABAergic system are associated with the pathogenesis of autism spectrum disorder (ASD). However, the mechanisms by which GABAergic system dysfunctions induce the pathophysiology of ASD remain unclear. We previously demonstrated that a selective type I 5α-reductase inhibitor SKF105111 (SKF) induced ASD-like behaviors, such as impaired sociability-related performance and repetitive grooming behaviors, in male mice. Moreover, the effects of SKF were caused by a decrease in the endogenous levels of allopregnanolone (ALLO), a positive allosteric modulator of the GABA_A_ receptor. In this study, we used SKF-treated male mice as a putative animal model of ASD and examined the effects of Kami-shoyo-san (KSS) as an experimental therapeutic strategy for ASD. KSS is a traditional Kampo formula consisting of 10 different crude drugs and has been used for the treatment of neuropsychiatric symptoms. KSS dose-dependently attenuated sociability deficits and suppressed an increase in grooming behaviors in SKF-treated mice without affecting ALLO content in the prefrontal cortex. The systemic administration of the dopamine D_1_ receptor antagonist SCH23390 reversed the ameliorative effects of KSS. On the other hand, the dopamine D_2_ receptor antagonist sulpiride and GABA_A_ receptor antagonist bicuculline only attenuated the ameliorative effect of KSS on repetitive self-grooming behaviors. The present results indicate that KSS improves SKF-induced ASD-like behaviors by facilitating dopamine receptor-mediated mechanisms and partly by neurosteroid-independent GABA_A_ receptor-mediated neurotransmission. Therefore, KSS is a potential candidate for the treatment of ASD.

## Introduction

Autism spectrum disorder (ASD) is a neurodevelopmental disorder involving diagnostic behavioral criteria such as social and communicative impairments and restrictive repetitive behaviors [1]. These symptoms often appear in the early stages of development [2] and are frequently comorbid with other diseases such as anxiety, depression, and attention deficit hyperactivity disorder (ADHD). Identified risk factors for ASD include genetic variations, epigenetic anomalies, and environmental factors [3], and the most critical risk factor for a cure are genetic variations. Hundreds of ASD-related genes have been detected, and mutations in these genes affect synaptic function, transcription factors, and neurotransmission [4–6].

Dysfunctions in the GABAergic system in the brain are associated with ASD symptoms. An analysis of post-mortem brain samples from ASD patients revealed that the expression levels of GABA_A_ and GABA_B_ receptors in the anterior cingulate cortex are decreased [7, 8]. Downregulation of GABAergic gene expression and reduced density of GABA-related proteins are also detected in animal models [9]. Moreover, GABAergic neurotransmission is decreased in the hippocampal CA1 region of BTBR mice, a model of idiopathic autism, and treatments with benzodiazepines improve deficits in social interactions, repetitive behaviors, and spatial learning [10]. However, it is still unclear how dysfunctions in GABAergic systems are linked to ASD symptoms.

Allopregnanolone (ALLO, 3α-hydroxy-5α-pregnan-20-one) is an endogenous neurosteroid that binds with high affinity to GABA_A_ receptors and exerts potent positive allosteric modulatory effects. ALLO is synthesized in the brain from progesterone by the activities of two enzymes, 5α-reductase type I, which reduces progesterone to 5α-dihydroprogesterone (5α-DHP), and 3α-hydroxysteroid dehydrogenase, which reduces 5α-DHP to ALLO or oxidizes ALLO to 5α-DHP [11]. The downregulation of neurosteroid biosynthesis contributes to the development of anxiety and depressive disorders, and reduced levels of allopregnanolone in peripheral blood or cerebrospinal fluid are associated with several affective disorders, including major depression, anxiety disorders, premenstrual dysphoric disorder, posttraumatic stress disorder, negative symptoms in schizophrenia, and impulsive aggression [12].

We previously demonstrated that social isolation rearing in the early post-weaning period resulted in a number of behavioral abnormalities in male mice, such as increased spontaneous physical activity, learning and memory deficits, attention deficits, and social deficits [13–16], which are symptoms observed in developmental disorders including ASD and ADHD. We also observed that social isolation stress decreased ALLO levels and mRNA and protein expression of 5α-reductase type I in the frontal cortex of mice [11, 17, 18]. Based on these findings, we speculated that ALLO may have a role in the regulation of developmental disorders, particularly ASD. A previous study conducted in our laboratory [19] confirmed that reductions in brain ALLO levels following the administration of SKF10511 (SKF), an inhibitor of type I 5α-reductase, a rate-limiting enzyme of ALLO biosynthesis, resulted in ASD-like behaviors in male mice. Thus, a decrease in brain ALLO levels may provide a potential animal model for investigating the pathophysiology of ASD and the development of new therapeutic strategies.

For the treatment of ASD-like behaviors caused by reduced ALLO content in the brain, we focused on Kami-shoyo-san (KSS), a traditional Japanese herbal medicine. KSS is a herbal formula that is clinically prescribed not only to attenuate menstrual irregularities, but also alleviates menstrual irregularity- or gynecological malignancy-related symptoms, such as anxiety, climacteric disturbances, and sleep disturbances [20–22]. KSS reportedly exerts antidepressant-like behavioral and neurochemical effects [23] as well as anxiolytic-like effects in male rodents [24]. In addition, the anxiolytic effects of KSS are involved in the neurosteroidal modulation of GABA_A_ receptors in the brain [24]. These pharmacological features of KSS prompted us to pre-clinically investigate whether KSS has potential for the treatment of ASD. Therefore, we examined the effects of KSS on ASD-like behavioral symptoms induced by a decreased biosynthesis of ALLO and pharmacologically elucidated the mechanisms underlying the effects of KSS.

## Materials and methods

### Animals

**Fig 1:**
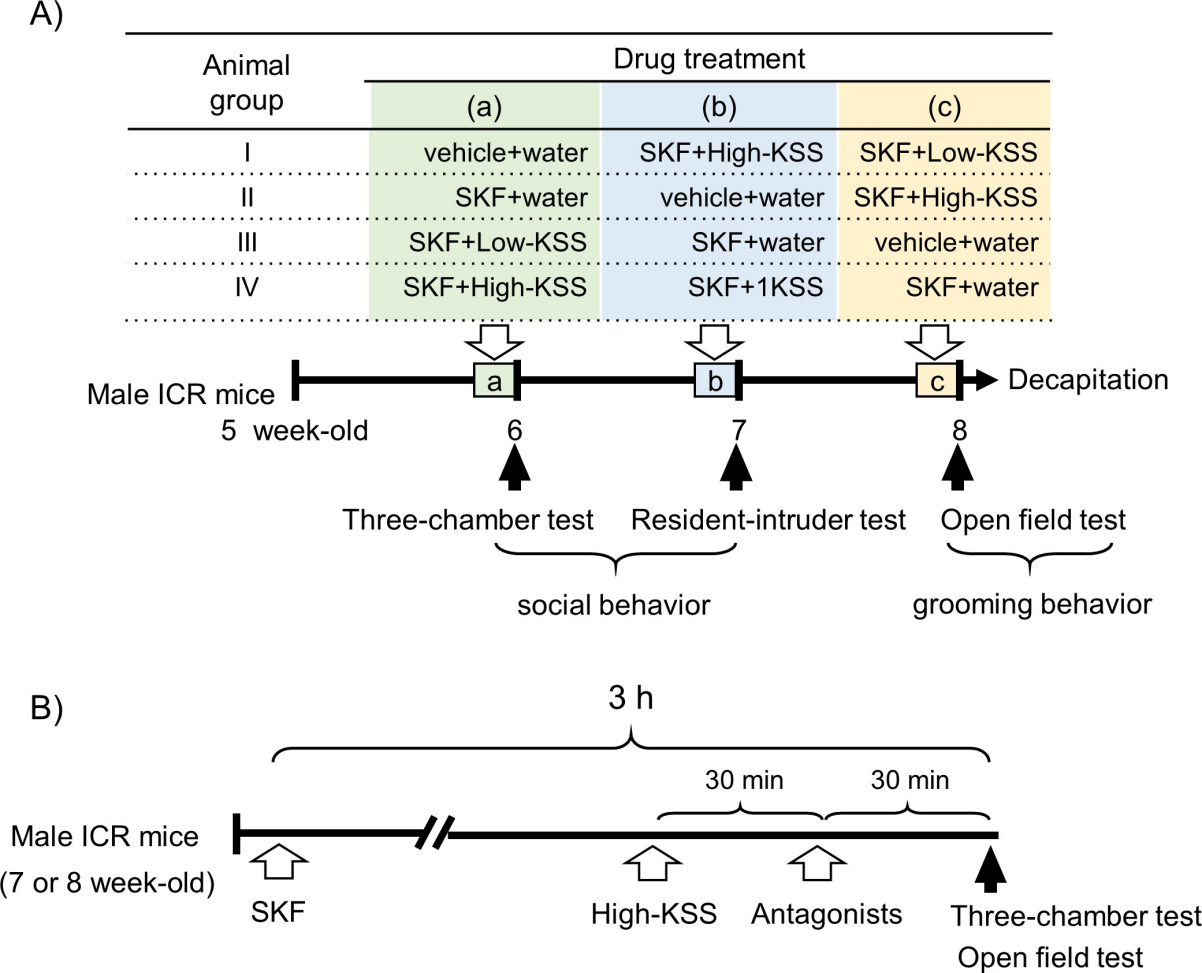
Experimental schedules. Experimental schedule A: A schedule to elucidate the effects of KSS on SKF-induced social affiliation deficits and grooming behaviors. Five-week-old ICR male mice were divided into 4 groups (I–IV). After a one-week acclimatization period, a three-chamber test, a resident-intruder test, and an open field test were conducted in a one-week period. Each animal group received vehicle + water, 40 mg/kg (i.p.) SKF + water (SKF + water), 74 mg/kg (p.o.) KSS (SKF+Low-KSS), or SKF + 222 mg/kg (p.o.) KSS (SKF+High-KSS) (a–c) before each behavioral test. After completing each test, the animals were returned to their home cages and left with no drug treatment until the next behavioral experiments. Experimental schedule B: A schedule to elucidate the effects of KSS pharmacologically using different receptor antagonists. The three-chamber test and open field test were conducted using 6-week-old and 8-week-old male mice, respectively. The animals received the test drug treatment according to the order indicated, and social affiliation behaviors and grooming behaviors were elucidated by a three-chamber test and an open field test, respectively. The animals were used only once in each behavioral experiment.

Male ICR mice (Japan SLC, Shizuoka, Japan) were obtained at the age of 5 weeks. Animals were housed in groups of 3–4 mice/cage (24 × 17 × 12 cm). The study was conducted according to the experimental schedules shown in Fig 1. Food and water were given *ad libitum*. Housing was thermostatically maintained at 24 ± 1°C with constant humidity (65%) and a 12-h light-dark cycle (lights on: 07:00–19:00). All animal research procedures were in accordance with the guiding Principles for the Care and Use of Animals (NIH Publications No. 80–23, revised in 1996). The present study was also approved by the Institutional Animal Use and Care Committee of the University of Toyama, Japan.

### Behavioral study

Three different behavioral tests were employed to analyze social affiliation behaviors and repeated grooming behaviors of mice. A three-chamber test and resident-intruder test were used to analyze social affiliation behaviors, and an open field test was used to analyze grooming behaviors. We examined the effects of 74 mg kg (Low-KSS) and 222 mg/kg KSS (High-KSS) on 40 mg/kg (i.p.) SKF-induced social affiliation deficits and grooming behaviors. The animals were divided into 4 groups, and each group was treated with vehicle+water, SKF+water, SKF+Low-KSS, or SKF+High-KSS in a semi-counterbalanced order before each behavioral test (Fig 1A). SKF and KSS were administered 3 h and 1 h before each behavioral test, respectively. After completing each test, the animals were returned to their home cages and left with no drug treatment until the next behavioral experiments.

We pharmacologically examined the effects of KSS using a three-chamber test and an open field test using 6- and 8-week-old male mice, respectively, according to the experimental schedule shown in Fig 1B. Receptor antagonists were injected (i.p.) 30 min before the behavioral tests according to the drug treatment protocol (Fig 1B). The animals were used only once in each test.

#### Sociability test

The social affiliation behavior was elucidated as an index of social behavior of mice using two experimental paradigms: a three-chamber test and resident-intruder test as previously described [16, 19, 25].

The three-chamber test: The equipment used in the three-chamber test included an open field [67 (W) × 14 (D) × 21 (H) cm] with gray walls and a black floor and was divided into 3 equal chambers by 2 opaque gates. Two identical empty Plexiglas cylinders (diameter: 10 cm and height: 18 cm) were placed on the sides of the equipment. The cylinder was transparent and had 45 small holes (0.9 cm in diameter) on the cylinder wall, permitting olfactory, visual, auditory, and some tactile contact, but no aggressive interactions for social affiliation as described previously [16]. The test consisted of a training trial and test trial. In the training trial, animals were individually placed in the center chamber of the equipment, and allowed to explore the arena freely for 5 min to acclimatize to the experimental arena and procedures, and then the test trial was conducted. In the test trial, a stranger mouse was placed into one of the cylinders as a social stimulus while the other cylinder remained empty. The cylinders and chambers were cleaned using 70% ethanol between trials to prevent a build-up of olfactory cues. Animal behavior during a 10-min observation period was video-recorded for later analysis. The social affiliation behavior and locomotor activity of each animal were analyzed automatically using SMART ver. 2.5 (PanLab, S.L., Barcelona, Spain) with a tri-wise module to detect the head, center mass, and base tail. Social affiliation was defined as subject mice spending more time in the stranger chamber with a stimulant mouse than in the chamber with an inanimate object [16, 19, 25, 26]; therefore, the time each mouse spent exploring a 2-cm width zone around the stranger and empty cylinder chambers during the observation period was measured as an index of social affiliation. To compare the social behaviors of each group, a discrimination index was calculated for each experimental group according to the following equation, where T represents the time that mice spent exploring each cylinder:

$$Discrimination\;index=\frac{T_{stranger}-T_{empty}}{T_{stranger}+T_{empty}}$$

Total distance animals traveled in the test trial was also measured using the Smart system (PanLab, S.L., Barcelona, Spain) as an index of locomotor activities.

The resident-intruder test: This experiment was conducted in a neutral home cage as described previously [14, 19, 25]. Briefly, 3 hours after treatment with vehicle or SKF, the treated mouse was placed in a neutral cage (24 × 17 × 12 cm) as a resident for 15 min. Then, an age- and sex-matched naïve mouse was placed in the cage as an intruder for 10 min. Social interactions between the resident and intruder animals were video-recorded, and the total cumulative duration of time the drug-treated resident mouse spent sniffing the intruder mouse was measured blindly using Microsoft Visual Basic-based software (EventRecord ver. 3). (Commentforreviewer1_8)

#### Open field test

An open field test was conducted to analyze both anxiety-related behavior and stereotyped grooming behavior as previously described [19, 27]. Briefly, mice were placed individually in an open field box (50 × 50 × 50 cm) with 1 lux lightness for 15 min. The first 10-min observation period was used to measure anxiety-like behavior, and the latter 10-min period between 5 and 15 min after starting the open field test was used to evaluate stereotyped grooming behavior. The activity of mice in the arena was video-recorded. The total duration of time each mouse spent in the center zone (30 × 30 cm square) was analyzed using SMART ver. 2.5. Anxiety-related behavior was expressed as a percentage of time spent in the center zone during the first 10-min observation period. The total duration of grooming behavior was analyzed by EventRecord ver. 3 as previously reported [19].

### Drug treatment

Drug treatments were conducted according to the experimental schedule described in Fig 1B. SKF105111 (SKF, 7h-(N,N-diisopropylcarbamoyl) and rost-3,5-diene-3-carboxylic acid), a type I 5α-reductase inhibitor, which was synthesized in the laboratory according to Holt et al. [28], were employed. Some pharmacological activities of SKF have been elucidated in previous studies [19, 29, 30]. SKF, which was processed before administration as described previously [19, 29, 30], was dissolved in 100% methanol and then mixed with an equimolar amount of NaOH to yield SKF-Na salt. SKF-Na (40 mg/kg) was dissolved in distilled water and injected intraperitoneally (i.p.) 3 h before behavioral experiments because SKF causes a significant decrease in brain ALLO content during this period [19, 29, 30]. The corresponding control group was administered the same volume of distilled water as a vehicle. Medicinal herbs were purchased from Tsumura Co. (TC: Tokyo, Japan) and Tochimoto tenkai-do (TT: Osaka, Japan) and conformed to the Japanese Pharmacopeia XVI guidelines. Kami-shoyo-san is a freeze-dried powder product made from a hot water extract of a mixture of 10 herbal ingredients: 3.0 parts *Bupleuri Radix* (Bupleurum Root, TC; lot number 24017551), 3.0 parts *Paeoniae Radix* (Peony Root, TT; lot number 005315011), 3.0 parts *Atractylodis Lanceae Rhizoma* (Atractylodes Lancea Rhizome, TC; lot number E38991), 3.0 parts *Angelicae Radix* (Japanese Angelica Root, TC; lot number K17591), 3.0 parts *Poria* (Poria Sclerotium, TT; lot number 009513010), 2.0 parts *Gardeniae Fructus* (Gardenia Fruit, TT; lot number 004613005), 2.0 parts *Moutan Cortex* (Moutan Bark, TT; lot number 010015005), 1.5 parts *Glycyrrhizae Radix* (Glycyrrhiza, TC; lot number 25001601), 1.0 part *Zingiberis Rhizoma* (Ginger, TT; lot number 005815002), and 1.0 part *Menthae Herba* (Mentha Herb, TT; lot number 008916004).

This powder was dissolved in tap water just before experiments, and administered orally (p.o.) 1 h before behavioral experiments at doses of 74 mg/kg (Low-KSS) and 222 mg/kg (High-KSS), which were equal to or approximately 3-fold higher than, respectively, the typical daily doses for human therapy. Each concentration of KSS was adjusted so that the administration volume was 0.1 mL. The corresponding control group was administered 0.1 mL of tap water as a vehicle. The chemical constituents of KSS were analyzed according to previous studies [25, 31] using a Shimadzu LC-IT-TOF mass spectrometer equipped with an ESI interface. The following ESI parameters were used: source voltage, +4.5 kV (positive ion mode) or −3.5 kV (negative ion mode); capillary temperature, 200°C; and nebulizer gas, 1.5 L/min. A Waters Atlantis T3 column (2.1 × 100 mm) was maintained at 40°C. The mobile phase was a binary eluent of (A) 5 mM ammonium acetate solution and (B) acetonitrile under the following gradient conditions: 0–50 min; linear gradient from 10% to 100% B, and 50–60 min; isocratic at 100% B. The flow rate was 0.2 mL/min. Mass spectrometry data obtained from KSS was stored in the Wakan-Yaku DataBase system, Institute of Natural Medicine, University of Toyama (http://dentomed.toyama-wakan.net/en/information_on_experimental_kampo_extracts/kamishoyosan%20extract-2018-KM/EXP008004). A voucher specimen of KSS (No. 20000009) was deposited at our institute. Other drugs were dissolved in distilled water and administered i.p. 30 min before the behavioral tests at the following doses: SCH23390 (SCH, 0.2 mg/kg), sulpiride (SUL, 5 mg/kg), bicuculline (BIC, 1 mg/kg), and phaclofen (PHA, 2 mg/kg). The corresponding control group was administered the same volume of distilled water as a vehicle.

### Enzyme-linked immunosorbent assay (ELISA) for ALLO content

An allopregnanolone enzyme-linked immunoassay kit (Arbor Assays, MI, USA) was used to measure ALLO content in brain tissue as previously described [19]. Brain tissues were dissected immediately after decapitation under mixed anesthesia (0.75 mg/kg medetomidine, 4 mg/kg midazolam, and 5 mg/kg butorphanol tartrate), and the frontal cortices were placed in liquid nitrogen and stored in a freezer at -80°C until use. ALLO was extracted from these tissues and quantified according to the manufacturer’s instructions. (Commentforeditor_2)

### Data analysis

Data analysis was conducted using SigmaPlot ver.12.3 (SYSTAT Software Inc., San Jose, CA, USA). In each experiment, comparison between the vehicle control group and the group administered only SKF was conducted by a Student's t-test or Mann-Whitney Rank Sum test, and comparison of the effects of KSS and antagonists among SKF administration groups was conducted by one-way ANOVA followed by the Student-Newman-Keuls test for multiple comparisons, or Kruskal-Wallis One Way Analysis of Variance on Ranks with a post-hoc Dunnett's test. Data obtained in the behavioral experiments (three-chamber test, resident-intruder test, and open field behaviors) were analyzed by the Mann-Whitney Rank Sum test for two groups or the Kruskal-Wallis test followed by Dunnett’s post-hoc test because the Shapiro–Wilk normality test for the data failed. ELISA data were analyzed by a Student’s *t*-test for two groups or a one-way ANOVA followed by the Student-Newman-Keuls test for multiple comparisons. Differences of P < 0.05 were considered significant. (Commentforreviewer2_9)

## Results

### Effects of KSS on SKF-induced ASD-like behaviors in mice

**Fig 2:**
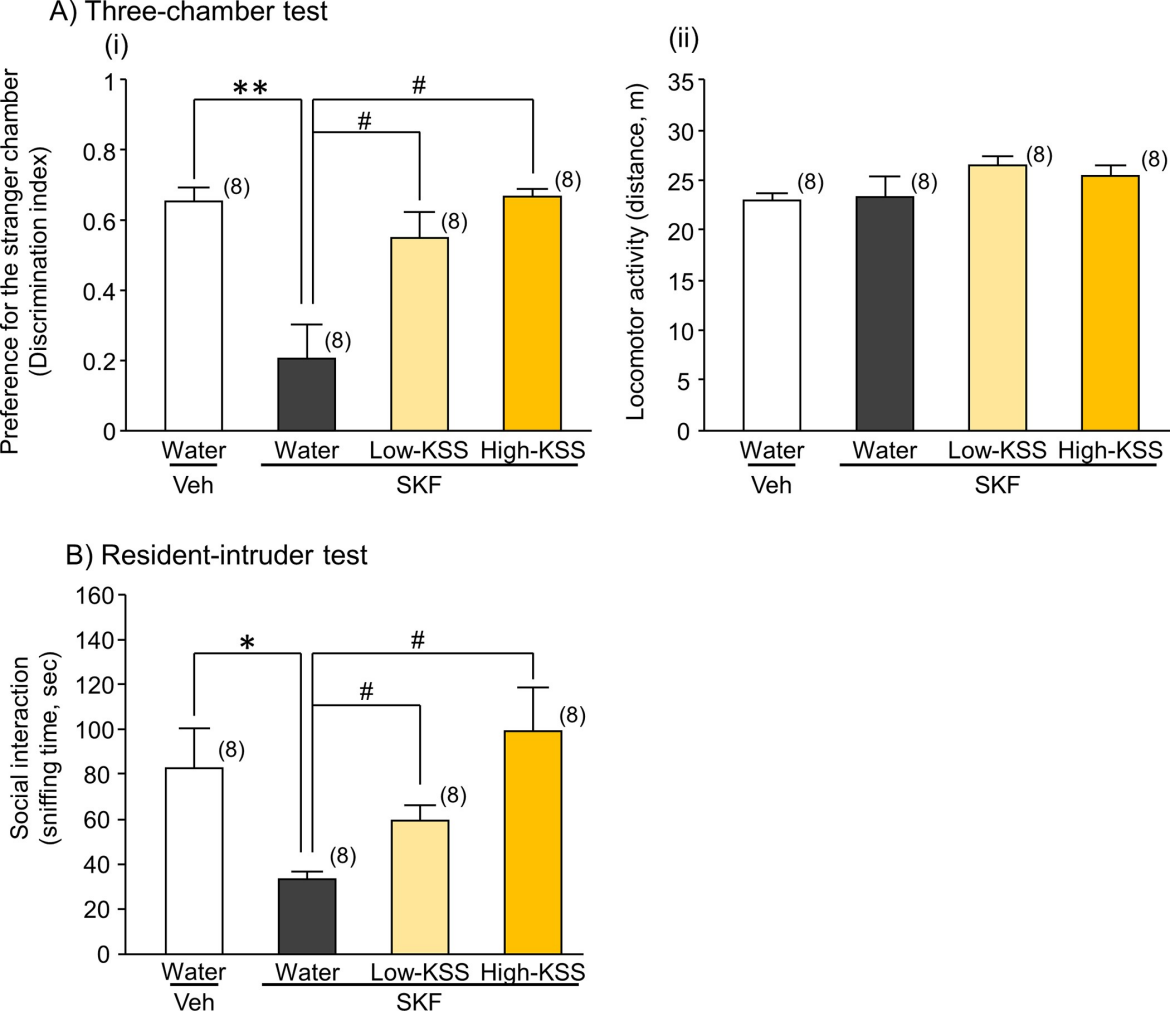
Effects of KSS on SKF-induced social deficits in male mice. The sociability-related performance of mice was analyzed by measuring social affiliation behaviors in the three-chamber test (A) and social interaction behaviors in the resident-intruder tests (B). A: Summarized data on social affiliation performance (i) and locomotor activities (ii) of each animal group in the test trials of the three-chamber test. The preference of each animal group for the stranger chamber was expressed as a discrimination index calculated according to the equation described in the text. Locomotor activity of each animal group in the test trial was measured using the Smart system. B: Social interactions between the drug-treated resident and naïve control animals. The total duration of the time the drug-treated resident mouse spent sniffing the intruder mouse was measured as an index of social interaction during a 10-min observation period. Vehicle or SKF (40 mg/kg, i.p.) was administered 3 hr before the behavioral test. SKF-treated animals were orally administered water or KSS [74 mg/kg (Low-KSS) and 222 mg/kg (High-KSS)] 1 hr before the test. Each data column represents the mean ± S.E.M. The numbers of animals used are indicated in each parenthesis. *P < 0.05, **P < 0.01 vs. the corresponding vehicle group. #P < 0.05 vs. SKF+water group.

We examined the effects of the administration of KSS on SKF-induced deficits in sociability-related performance in male mice using the three-chamber and resident-intruder tests (Fig 2). In the three-chamber test, the preference for the stranger chamber of SKF-treated mice was reduced compared to that of the vehicle group (U = 1.000, P < 0.001). The effects of SKF treatment on sociability-related performance in the three-chamber test were significantly attenuated in the animal groups administered KSS [H(2) = 13.040, P = 0.001] in a concentration-dependent manner (Low-KSS: P < 0.05, High-KSS: P < 0.05) (Fig 2Ai). None of the treatments with SKF alone or SKF+KSS affected the locomotor activities of the animals in this test [Veh control vs. SKF: U = 25.000, P = 0.505; SKF treatment group: H(2) = 2.240, P = 0.326] (Fig 2Aii). Moreover, KSS did not affect the sociability-related performance of SKF-untreated mice (U = 30.000, P = 0.878, S1A and S1B Fig).

The effects of KSS on sociability-related performance were also confirmed by a resident-intruder test (Fig 2B). SKF-treated mice spent significantly less time sniffing the intruder mice, a novel social target (U = 8.000, P = 0.010), and this impairment was improved by the administration of KSS [H(2) = 8.375, P = 0.015] in a concentration-dependent manner (Low-KSS: P < 0.05, High-KSS: P < 0.05) (Fig 2B). Under the experimental conditions, which included 15 minutes of habituation time for the resident mouse prior to the introduction of the intruder, aggressive behaviors of resident mice in all groups were not observed.

**Fig 3:**
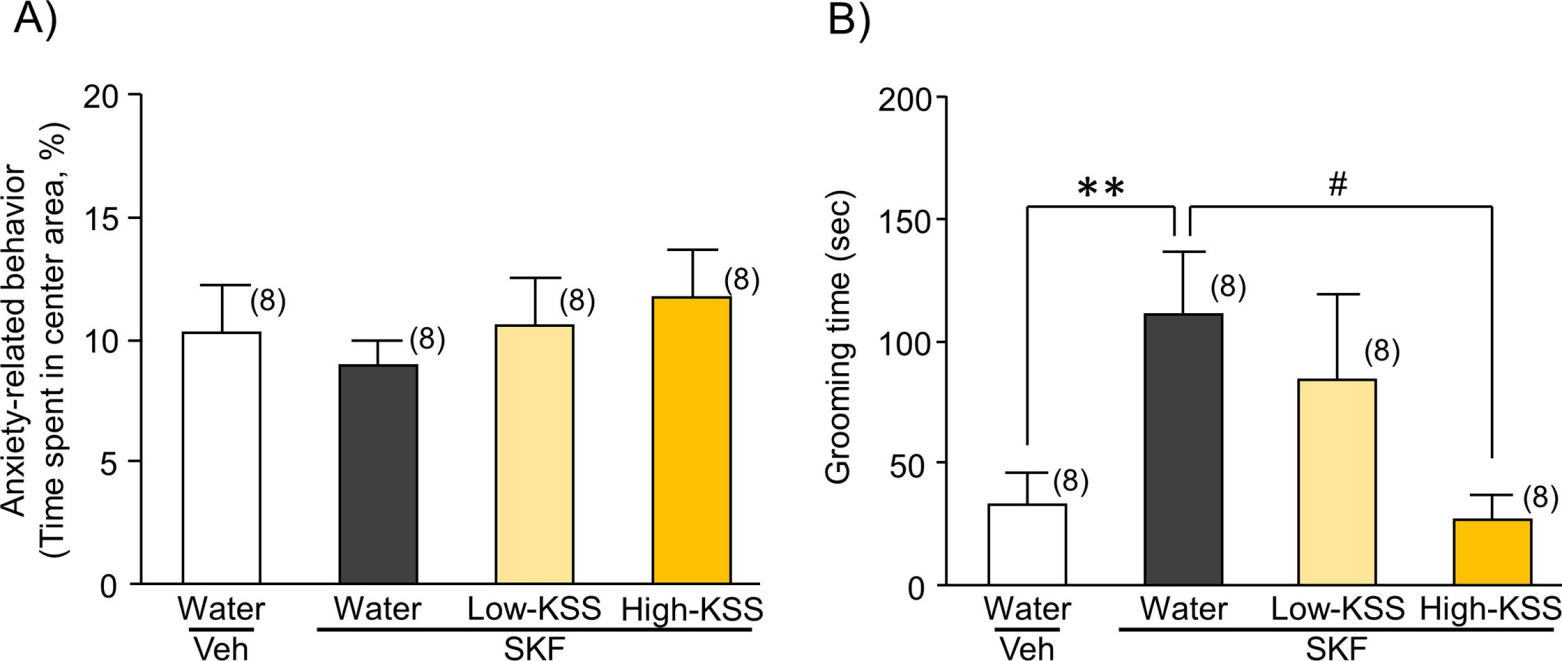
**Effects of KSS on anxiety-related behaviors (A) and repetitive grooming behaviors (B) in SKF-treated mice. A)** Anxiety-like behaviors of each mouse group were analyzed as the percentage of time spent in the center zone in the open field during the first 10-min observation period. **B)** The total cumulative duration of each mouse spent for self-grooming was measured during the latter 10-min observation period between 5 and 15 min after starting the open field test. KSS was administered orally at doses of 74 mg/kg (Low-KSS) and 222 mg/kg (High-KSS) 1 h before the test. Each data column represents the mean ± S.E.M. The numbers of animals used are indicated in each parenthesis. **P < 0.01 vs. the corresponding vehicle group. #P < 0.05 vs. SKF+water group.

We then conducted an open field test to investigate the effects of KSS on SKF-induced self-grooming behavior and activity in the center area, as indices of restrictive repetitive behavior and anxiety-related behavior, respectively. The administration of SKF to mice significantly increased self-grooming times without significantly affecting exploration times in the center arena of the open field (self-grooming time: U = 6.000, P = 0.005; anxiety-related behavior: U = 25.000, P = 0.505) (Fig 3A and 3B). Moreover, KSS reversed the SKF-induced increase in self-grooming behaviors [H(2) = 8.880, P = 0.012] by the administration of High-KSS (P < 0.05) (Fig 3A). KSS did not affect the self-grooming behavior of SKF-untreated mice as well as the result of the three-chamber test (U = 29.000, P = 0.798, S1A and S1C Fig).

### Effects of KSS on SKF-induced decreases in ALLO content in the brain

**Fig 4:**
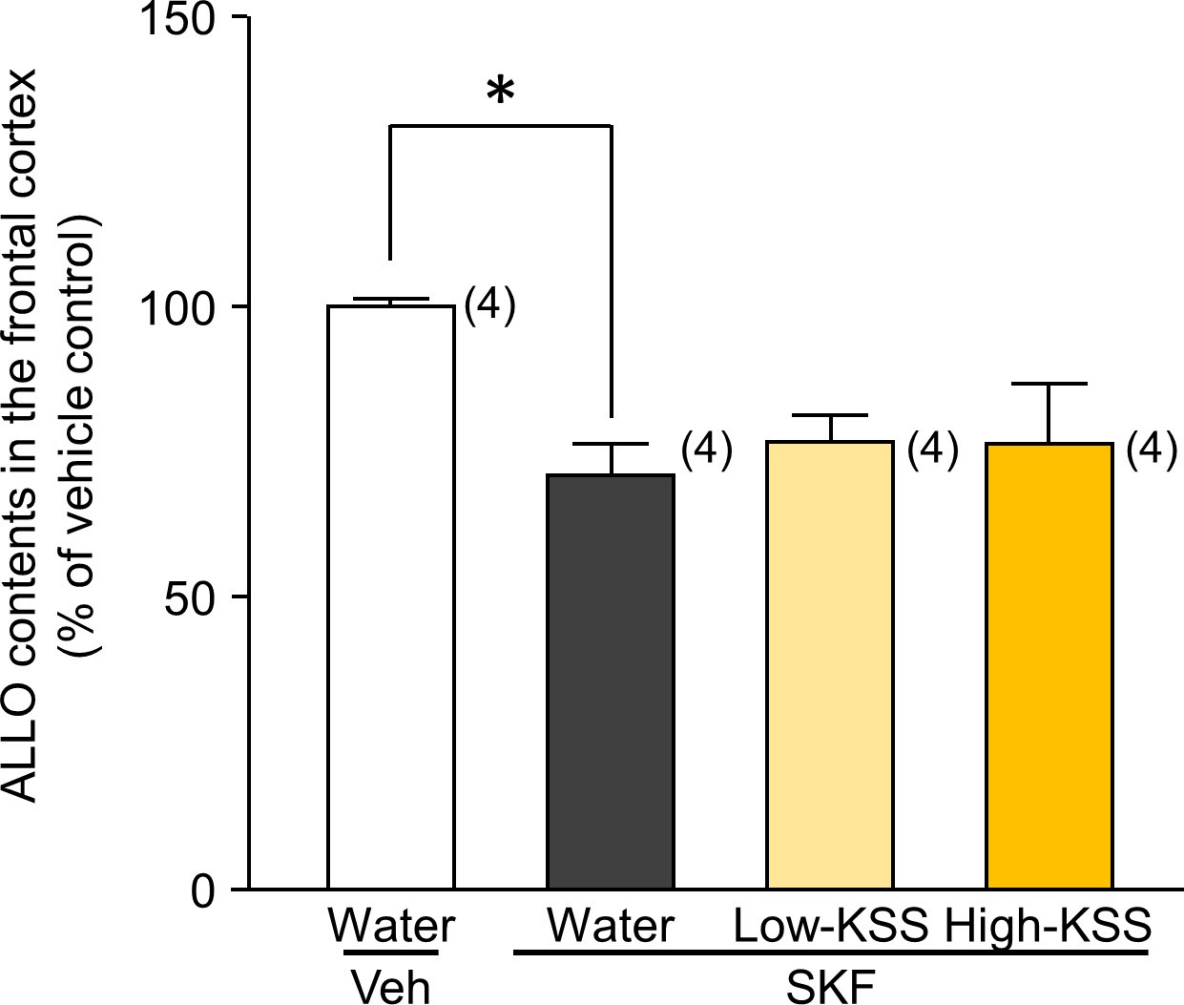
Effects of KSS on SKF-induced decrease in endogenous ALLO content in the frontal cortex. After completing the open field test conducted according to experimental schedule A (Fig 1), each animal was decapitated, and the frontal cortices were dissected for neurochemical studies. ALLO content in the frontal cortex was measured using ELISA. Each data column represents the mean ± S.E.M., and the numbers of animals used are indicated in each parenthesis. *P < 0.05 vs. the corresponding vehicle group.

We examined whether the ameliorative effects of KSS on SKF-induced ASD-like behaviors were caused by changes in ALLO levels in SKF-treated animals by measuring brain ALLO content with allopregnanolone ELISA. The ELISA results revealed that SKF treatment significantly reduced ALLO content in the frontal cortex [t(6) = 2.978, P = 0.025], whereas SKF-induced decreases in ALLO content were not reversed by the administration of either dose of KSS [F(2,9) = 0.0814, P = 0.922] (Fig 4).

### Involvement of dopaminergic and GABAergic mechanisms on the ameliorative effects of KSS on SKF-induced ASD-like behaviors in mice

**Fig 5:**
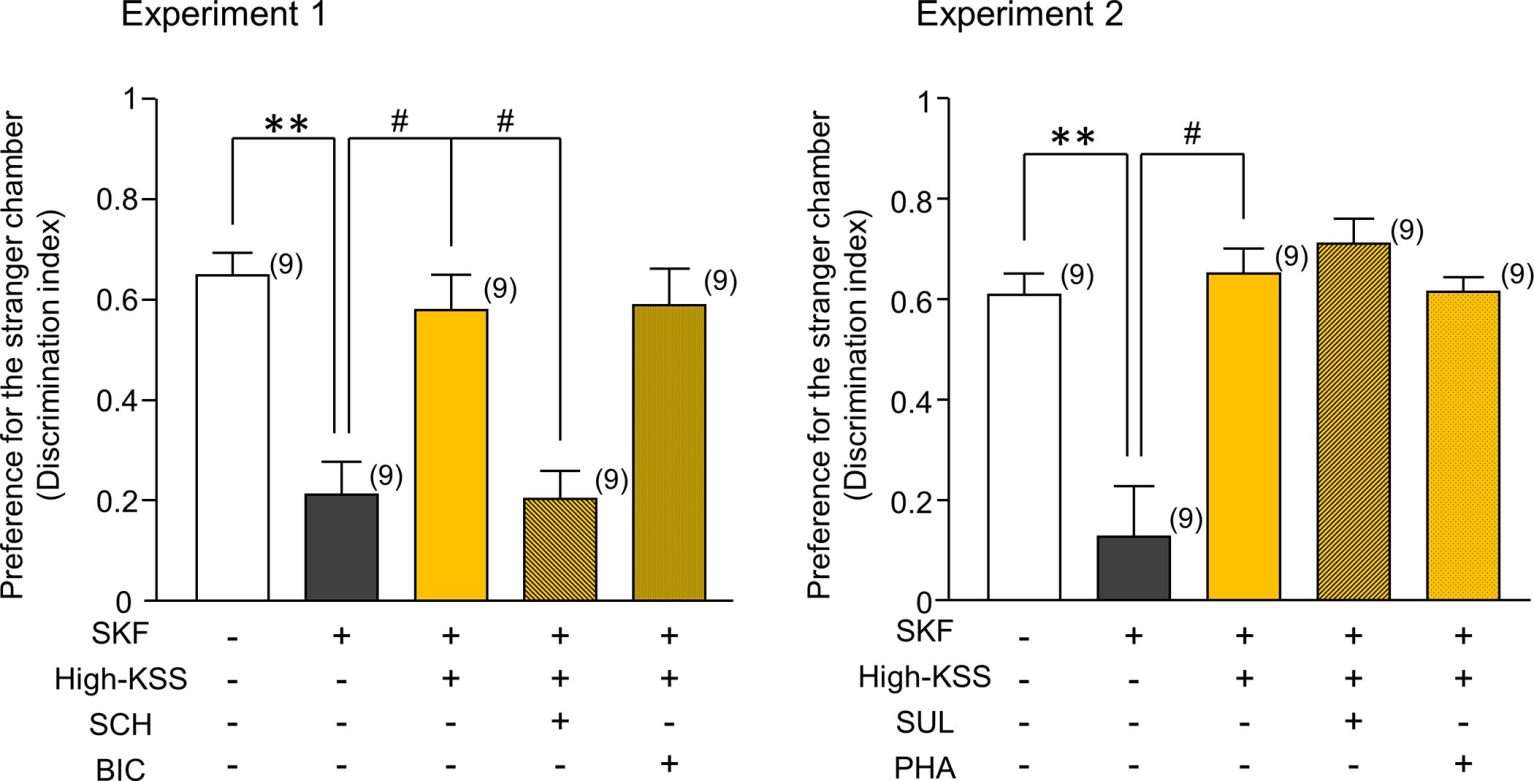
Pharmacological analysis of the ameliorative effects of KSS on SKF-induced social deficits in male mice. Preference for the stranger chamber was expressed as a discrimination index and used as an index of sociability-related performance in mice. A discrimination index was calculated according to the equation described in the text. Experiments 1 and 2 were conducted using different animal groups. Antagonists [SCH23390 (SCH: 0.2 mg/kg), sulpiride (SUL: 5 mg/kg), bicuculline (BIC: 1 mg/kg), phaclofen (PHA: 2 mg/kg)] were injected intraperitoneally 30 min before the test. Each data column represents the mean ± S.E.M., and the numbers of animals used are indicated in each parenthesis. **P < 0.01 vs. the corresponding vehicle group. #P < 0.05 vs. SKF+High-KSS group.

To elucidate the mechanisms underlying the effects of KSS, we investigated the possible involvement of dopaminergic and GABAergic mechanisms on the effects of KSS on episodes of ASD-like behaviors caused by SKF. As shown in Fig 5A and 5B, there were significant changes in SKF-induced sociability deficits after High-KSS and antagonist treatments [Fig 5A: H(3) = 15.513, P = 0.001; Fig 5B: H(3) = 17.190, P < 0.001]. The ameliorative effects of KSS on SKF-induced deficits in sociability-related performance were almost completely abolished by the administration of SCH23390, a selective dopamine D_1_ receptor antagonist (P < 0.05), but not by sulpiride, a selective dopamine D_2_ receptor antagonist (Fig 5A and 5B). Based on the pharmacological feature of ALLO as a positive allosteric modulator of the GABA_A_ receptor, we also examined whether the GABAergic system was involved in KSS in SKF-treated mice using the GABA_A_ receptor antagonist bicuculline and the GABA_B_ receptor antagonist phaclofen. Neither bicuculline nor phaclofen affected the High-KSS-induced improvement in sociability-related performance impaired by SKF treatment in the three-chamber test (Fig 5A and 5B). These findings suggest that KSS improves SKF-induced sociability deficits via facilitation of a dopamine D_1_-dependent mechanism.

**Fig 6:**
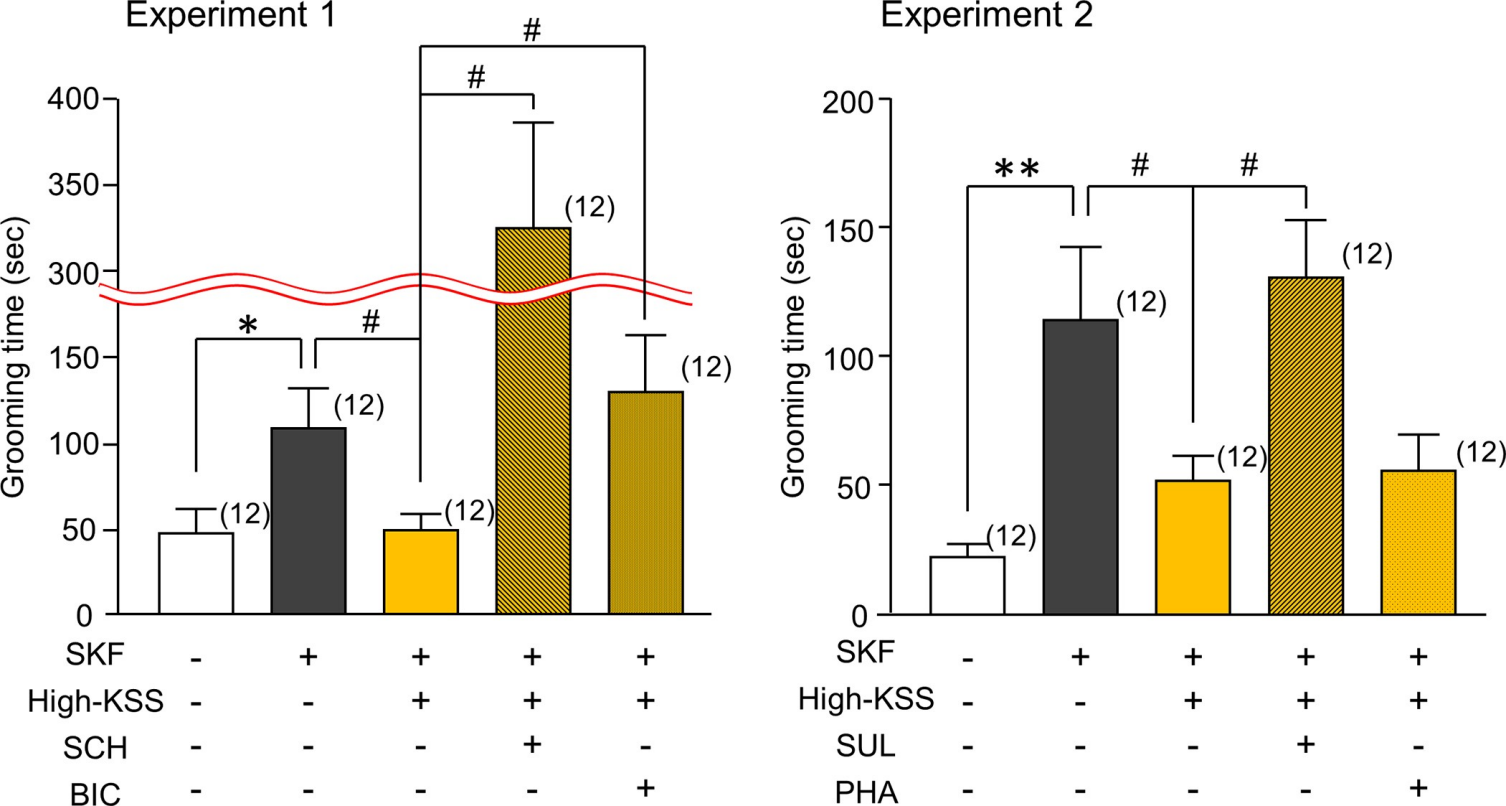
Pharmacological analysis of the ameliorative effects of KSS on SKF-induced repetitive self-grooming behaviors in male mice. Time each animal spent for repetitive self-grooming was measured in an open field test. Experiments 1 and 2 were conducted using different animal groups. Antagonists [SCH23390 (SCH: 0.2 mg/kg), sulpiride (SUL: 5 mg/kg), bicuculline (BIC: 1 mg/kg), phaclofen (PHA: 2 mg/kg)] were injected intraperitoneally 30 min before the test. Each data column represents the mean ± S.E.M., and the numbers of animals used are indicated in each parenthesis. *P < 0.05, **P < 0.01 vs. the corresponding vehicle group. #P < 0.05 vs. SKF+High-KSS group.

In the open field test, there were significant changes in SKF-induced grooming behavior after High-KSS and antagonist treatments [Fig 6A: H(3) = 17.190, P < 0.001; Fig 6B: H(3) = 11.016, P = 0.012]. The suppression of SKF-induced repeated self-grooming behavior by High-KSS was significantly abolished by SCH23390 (P < 0.05), sulpiride (P < 0.05), and bicuculline (P < 0.05) (Fig 6A and 6B). However, the effects of High-KSS on SKF-induced grooming behavior did not appear to be affected by the administration of phaclofen. These findings suggested that High-KSS improved SKF-induced repetitive self-grooming behaviors via facilitation of dopaminergic mechanisms and in part via GABA_A_ receptor-mediated neurotransmission.

## Discussion

We demonstrated that KSS improved ASD-like behaviors in SKF-treated male mice via the stimulation of dopaminergic and, partly, GABA_A_ receptor mechanisms in the brain. These results not only support our hypothesis that ASD-like behaviors caused by a decrease in brain ALLO content are caused by an impaired link between the GABAergic and dopaminergic systems [19], but also provide a new potential therapeutic strategy for ASD using Kampo medicine.

Treatment of male mice with SKF, a type I 5α-reductase inhibitor, caused ASD-relevant behaviors such as impaired sociability-related performance and an increased level of repeated grooming behavior. These ASD-related behaviors exhibited by SKF-treated animals were consistent with our previous findings [19] showing that SKF downregulates brain ALLO biosynthesis and induces ASD-like behaviors in a manner that is reversible by exogenous ALLO and methylphenidate (MPH), a dopamine transporter inhibitor. Dysfunctions in the GABAergic system are involved in the pathophysiology of ASD [7, 32]. Therefore, we hypothesized that impaired ALLO biosynthesis in a sex-dependent manner may be an underlying mechanism of ASD and provide a rational animal model to explore effective therapeutic strategies for ASD.

We focused on KSS, a traditional Japanese Kampo medicine, and examined whether its administration affected ASD-like symptoms. KSS reportedly exerts anxiolytic effects via neurosteroidal modulation of GABA_A_ receptor mechanisms [24]. We found that KSS dose-dependently improved sociability deficits in SKF mice in a three-chamber test and resident-intruder test without affecting motor activity or anxiety-related performance in an open field test. We also revealed that KSS did not affect the sociability-related performance itself of mice in the 3-chamber test. These results are consistent with our previous findings [19] showing that SKF treatment impairs sociability more selectively than it exacerbates stress-induced anxiety-like behavior via a decrease in endogenous brain ALLO content. Moreover, the present results suggest that the administration of KSS preferentially improves sociability deficits caused by SKF treatment.

KSS had no effect on the SKF-induced decline in ALLO content in the brain. The failure of KSS to affect brain ALLO content in SKF-treated animals is in contrast to previous findings reported by Mizowaki et al. [24] and indicates that the improvement in SKF-induced ASD-like behaviors by KSS occurred in a manner independent of ALLO. We previously suggested that ALLO regulates episodes of ASD-like behavior by positively modulating the function of GABA_A_ receptors linked to the dopaminergic system [19]. Collectively, these findings and the present results indicate that the ameliorative effects of KSS on ASD-like behaviors were attributable to the downstream cascade of ALLO, which includes the direct activation of GABA_A_ receptors, the facilitation of dopaminergic systems, or both.

The ameliorative effects of KSS on SKF-induced sociability deficits were completely abolished by a selective dopamine D_1_ receptor antagonist SCH23390, but not by a dopamine D_2_ receptor antagonist sulpiride. The GABA_A_ receptor antagonist bicuculline and the GABA_B_ receptor antagonist phaclofen did not alter the effects of KSS on SKF-induced sociability deficits. We previously reported that the administration of MPH, a dopamine transporter inhibitor, attenuated the impaired sociability performance observed in socially isolated mice and that the effects of MPH were blocked by a dopamine D_1_ receptor antagonist, but not by a dopamine D_2_ receptor antagonist, indicating an important role for dopamine D_1_ receptors in the regulation of sociability performance [16]. Moreover, MPH attenuated sociability deficits caused by SKF in male mice. Therefore, KSS and MPH appear to enhance dopaminergic systems, particularly the function of dopamine D_1_ receptors, which may be downstream of GABAergic systems in the brain, thereby improving the sociability performance of SKF-treated mice. This concept appears to be supported by previous findings. Nguyen et al. reported that the central dopaminergic system is closely involved in the pathogenesis of ASD, including genes encoding dopamine receptors, dopamine transporters, and enzymes for dopamine biosynthesis and catabolism [33]. Furthermore, MPH reportedly improved social interaction deficits in a prenatal valproic acid-treated mouse model of ASD through dopamine D_1_ and D_2_ receptors [34]. Therefore, KSS may improve sociability deficits in SKF-treated male mice by promoting dopaminergic systems at the level of dopamine D_1_ receptors.

One of the interesting results in the present study is that KSS, at the same dose that improved sociability deficits (222 mg/kg), significantly attenuated SKF-induced repetitive self-grooming behaviors without changing normal level grooming behavior. Furthermore, the effects of 222 mg/kg KSS were susceptible to the GABA_A_ receptor antagonist bicuculline but not susceptible to the GABA_B_ receptor antagonist phaclofen. Thus, based on the different susceptibilities to GABA receptor antagonists, the effects of KSS may be mediated, in part, by the direct stimulation of GABA_A_ receptors. This appears to be supported by an *in vitro* study conducted by Sugiyama et al. showing that KSS includes three herbal ingredients *Bupleuri radix*, *Zingiberis rhizome*, and *Paeoniae radix*, which have the potential to activate GABA_A_ receptors [35].

The effects of KSS on grooming behaviors were completely blocked not only by the selective dopamine D_1_ receptor antagonist SCH23390 but also by the dopamine D_2_ receptor selective antagonist sulpiride, which indicates that the ameliorative effects of KSS on repetitive grooming behaviors caused by SKF were caused by dopamine D_1_ and D_2_ receptor mechanisms. Because the present results showed that the ameliorative effects of KSS on SKF-induced sociability deficits were susceptible to SCH23390, but not sulpiride, it is likely that the dopaminergic system regulating repetitive grooming behaviors differs from the dopaminergic system involved in sociability-related performance. This idea conflicts with a recent report by Lee et al. [36]. They showed that knockout or knockdown of the dorsal striatal D_2_ dopamine receptors promoted ASD-like behaviors, such as sociability deficits and repetitive grooming behaviors, via inducing excessive D_1_ dopamine receptor activation. The reason for this conflict regarding the role of D_1_ and D_2_ dopamine receptors in ASD-like behaviors is unclear. We previously reported that MPH, a dopamine transporter inhibitor, attenuated not only sociability deficits but also repeated grooming behaviors in SKF-treated male mice [19]. Thus, taken together, the present results suggest that the administration of KSS can enhance the functions of two different dopaminergic systems involved in the regulation of sociability performance and repeated grooming behaviors. The molecular mechanisms underlying the faciliatory effects of KSS on the dopaminergic systems in the brain have not been elucidated in detail, but a speculative explanation is that KSS includes chemical ingredients with the potential to facilitate dopaminergic transmission in the brain. To clarify such chemical ingredients involved in the effect of KSS requires further investigation.

The present study revealed that SCH23390 (0.2 mg/kg, i.p.) significantly enhanced SKF-induced repetitive grooming behaviors in an animal group treated with KSS. The mechanism by which SCH23390 treatment exacerbated the grooming behaviors in this animal group was unclear but may be related the dose of SCH23390 used. The effects of SCH23390 on grooming behaviors in rodents are biphasic depending on the dose tested; low doses of SCH23390 cause a small increase in grooming behaviors in mice and rats [37–39].

In conclusion, the present study demonstrated that KSS at the same concentration as a typical daily dose for human therapy improved SKF-induced ASD-like behaviors via dopaminergic mechanisms and, in part, by a neurosteroid-independent GABA_A_ receptor mechanism. Moreover, our results demonstrate the potential of KSS in the treatment of ASD.

## Supporting information

S1 File Dataset of all experiments. (XLSX)

S1 Fig Effects of KSS on the sociability-related performance and grooming behavior of SKF-untreated male mice. (A) Experimental schedule: A schedule to elucidate the effects of KSS on the sociability-related performance and grooming behavior of SKF-untreated male mice. Six-week-old ICR male mice were divided into 2 groups (I–II). After a one-week acclimatization period, a three-chamber test and an open field test were conducted in a one-week period. Each animal group received water or 222 mg/kg (p.o.) KSS (High-KSS) (a–b) before each behavioral test. After completing each test, the animals were returned to their home cages and left with no drug treatment until the next behavioral experiments. (B) The sociability-related performance of mice was analyzed by measuring social affiliation behaviors in the three-chamber test. The preference of each animal group for the stranger chamber was expressed as a discrimination index calculated according to the equation described in the text. (C) The total cumulative duration of each mouse spent for self-grooming was measured during the latter 10-min observation period between 5 and 15 min after starting the open field test. KSS was administered orally at doses of 222 mg/kg (High-KSS) 1 h before the test. Each data column represents the mean ± S.E.M. The numbers of animals used are indicated in each parenthesis.
(TIF)
