# Supplementary figures and images for: Kami-shoyo-san improves ASD-like behaviors caused by decreasing allopregnanolone biosynthesis in an SKF mouse model of autism
Source: PLoS One. 2019 Jan 31;14(1):e0211266. doi: 10.1371/journal.pone.0211266 (PMC6354989; doi:10.1371/journal.pone.0211266)

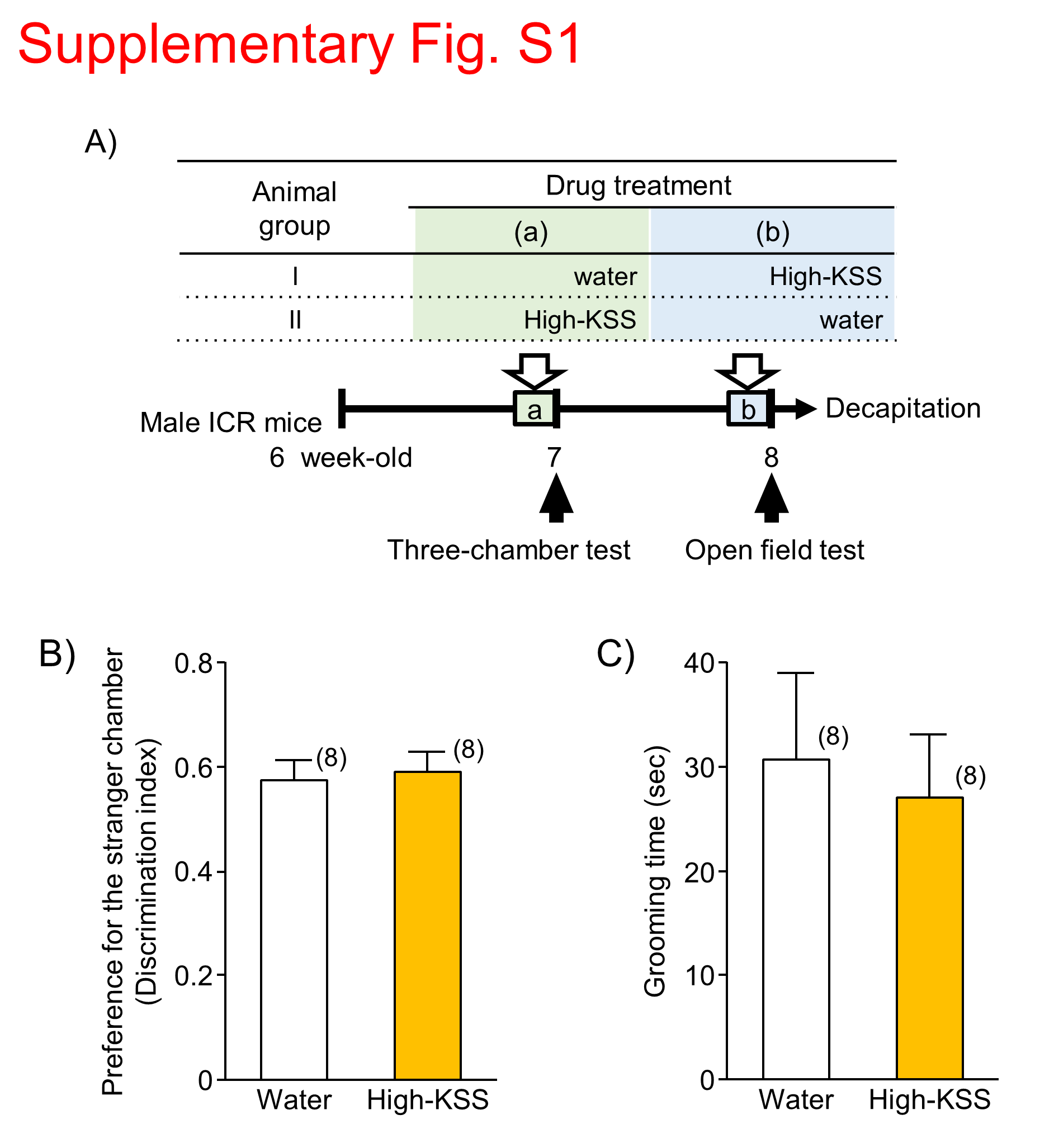

Supplement: S1 Fig — (A) Experimental schedule: A schedule to elucidate the effects of KSS on the sociability-related performance and grooming behavior of SKF-untreated male mice. Six-week-old ICR male mice were divided into 2 groups (I–II). After a one-week acclimatization period, a three-chamber test and an open field test were conducted in a one-week period. Each animal group received water or 222 mg/kg (p.o.) KSS (High-KSS) (a–b) before each behavioral test. After completing each test, the animals were returned to their home cages and left with no drug treatment until the next behavioral experiments. (B) The sociability-related performance of mice was analyzed by measuring social affiliation behaviors in the three-chamber test. The preference of each animal group for the stranger chamber was expressed as a discrimination index calculated according to the equation described in the text. (C) The total cumulative duration of each mouse spent for self-grooming was measured during the latter 10-min observation period between 5 and 15 min after starting the open field test. KSS was administered orally at doses of 222 mg/kg (High-KSS) 1 h before the test. Each data column represents the mean ± S.E.M. The numbers of animals used are indicated in each parenthesis. (TIF) [file pone.0211266.s002.tif]
